# Development of In Situ Microfluidic System for Preparation of Controlled Porous Microsphere for Tissue Engineering

**DOI:** 10.3390/pharmaceutics14112345

**Published:** 2022-10-30

**Authors:** Ji Hwan Han, Chul Min Kim, Tae-Hyun Kim, Songwan Jin, Gyu Man Kim

**Affiliations:** 1 School of Mechanical Engineering, Kyungpook National University, 80 Daehakro, Daegu 41566, Korea; 2 Department of Mechatronics Engineering, Gyeongsang National University, 33 Dongjin-ro, Jinju 52725, Korea; 3 R&D Center, Tissue Engineering Bio Science, 194-41, Osongsaengmyeong 1-ro, Cheongju-si 28159, Korea; 4 Department of Mechanical Engineering, Tech University of Korea, 237 Sangidaehak-ro, Siheung-si 15073, Korea

**Keywords:** microfluidics, porous microsphere, controlled pore, cell delivery, inflammation

## Abstract

In this study, we present an in situ microfluidic system to precisely control highly porous polycaprolactone microspheres as tissue templates for tissue engineering. The porosity of the microspheres was controlled by adjusting the flow rates of the polymer phase and the pore-generating material phase in the dispersed phase. The microfluidic flow-focusing technique was adopted to manufacture porous microspheres using a relatively highly viscous polymer solution, and the device was fabricated by conventional photolithography and PDMS casting. The fabricated in situ microfluidic system was used to precisely control the pore size of monodispersed polycaprolactone microspheres. The porous microspheres with controlled pore sizes were evaluated by culturing HDF cells on the surface of porous microspheres and injection into the subcutaneous tissue of rats. We found that the increased pore size of the microspheres improved the initial proliferation rate of HDF cells after seeding and relieved the inflammatory response after the implantation of porous microspheres in the subcutaneous tissue of rats.

## 1. Introduction

Porous microspheres have the potential to be used as injectable substrates for drug delivery because of their high surface area, excellent circulation with the surrounding environment, and precultured cell protection from physical damage [1,2,3]. They have attracted attention in various fields, such as biology, pharmacy, and medical sciences, especially in regeneration engineering [1,2,3,4,5].

The uniformity of injectable porous microspheres is essential to control the release properties of loaded functional materials [6,7]. Therefore, the improvement of the uniformity of porous microspheres has been extensively investigated [8]. Microfluidic systems can provide precise size control and narrow size distribution of porous microspheres, which are required for potential applications [8,9,10]. Furthermore, microfluidic systems can enable precise linear control over functional material loading and pre- and post-treatments in real time in combination with other microfluidic systems [11,12].

As cover the current state-of-the-art, Duncanson et al. proposed approach to fabricated porous microsphere with nano pore generated from microbubbles by perfluorinated-dendrimer–dye complex [4]. Yu et al. fabricated hollow cellulose microsphere by removing sacrificial material in microsphere for wound healing [5]. However, in these approaches, the pore sizes of porous microspheres have been predetermined by the premixing process between the polymer phase and the pore-generating material phase before injection into the microfluidic device, limiting the dispersibility of the pore-generating material phase in the polymer solution during the generation of porous microspheres [4,7,13,14]. In addition, these systems do not have the flexibility to control the pore size of porous microspheres.

To overcome these problems, in situ microfluidic systems can be used to prepare mixed microdroplets of a polymer and a pore-generating material in a microchannel without a premixing process [15,16]. The polymer and the pore-generating material solution are coloaded for the formation of microdroplets and mixed homogenously during their flow in the microchannel [15,16]. The homogeneous dispersion of the pore-generating material in microdroplets can be maintained, and the pore sizes of porous microspheres can be precisely controlled by controlling the flow rate ratios of the polymer and the pore-regenerating material. This mechanism would improve the mixing of the polymer and the pore-regenerating material to produce porous microspheres with controlled pore size. To the best of our knowledge, this is the first attempt to control the pore size of highly porous microspheres using an in situ microfluidic system.

Camphene (CAMP) has been widely used as a pore-generating material to prepare porous structures. It has a drastically increased volume in the solid phase after polymerization by evaporation. Therefore, highly porous structures in microspheres can be prepared by the reduced volume of polymer and highly increased volumes of CAMP during solvent evaporation [14,17,18]. CAMP is not only nontoxic and environmentally friendly but also soluble in organic solvents with low viscosity [14,17,18]. Owing to these properties, they can be easily adopted as pore-generating materials in in situ microfluidic systems. Polycaprolactone (PCL) has been used for the fabrication of porous microspheres in microfluidic systems because it is a biocompatible and degradable polymer and its use has been approved by the Food and Drug Administration (FDA) [19,20]. Their porous microspheres have great potential to be utilized in various medical applications [19,20].

In this study, we propose an in situ microfluidic system to prepare highly porous PCL microspheres with controlled pore sizes. The in situ microfluidic device was fabricated through conventional photolithography and soft lithography. The pore size of the microspheres was controlled by the flow rate ratios of the polymer and the pore-generating material phases. The diameter and pore size of porous microspheres were measured to evaluate the performance of the in situ microfluidic system. In vivo and in vitro experiments were conducted to observe the pore size effect of the porous microspheres. HDF cells were cultured on the porous microspheres, and the proliferation of HDF cells was analyzed by the CCK assay. Additionally, to inspect the pore size effect, three porous microsphere samples of different pore sizes were injected into the subcutaneous tissue of rats, and the tissue samples were analyzed after 4 weeks with histological and immunohistochemical assays.

## 2. Materials and Methods

### 2.1. Design of Microfluidic Flow

**Figure 1:**
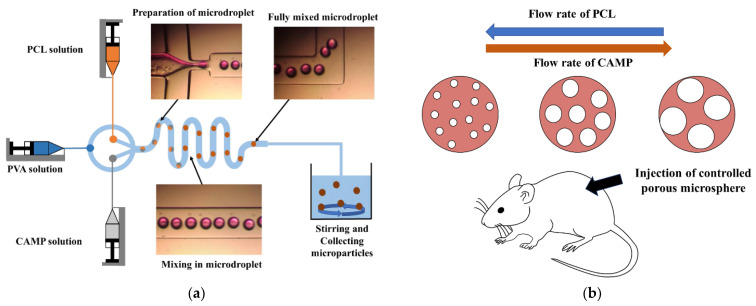
The generation process of highly porous microspheres with controllable pores by the in situ microfluidic system: (**a**) total microfluidic system, (**b**) mechanism for controlling pores of highly porous microspheres by adjusting flow rates and animal test.

Figure 1 shows the generation process of highly porous PCL microspheres with a controllable pore size by the in situ microfluidic system. The PCL (polycaprolactone, MW 80,000, Sigma Aldrich, Seoul, South Korea) and CAMP (camphene, MW 136, Sigma Aldrich, Seoul, South Korea) solutions were used as the dispersed phase, and the polyvinyl alcohol (PVA, MW: 30,000–70,000, Sigma Aldrich, Seoul, South Korea) solution was used as the continuous phase. They were injected into the in situ microfluidic device and controlled by a syringe pump independently (Pump 11 Elite, Harvard Apparatus, Holliston, MA, USA). The PCL and CAMP solutions flowed parallel to each other in the same microchannel, and the microdroplets involving the coloaded PCL and CAMP solutions were generated at the orifice by the shear stress of the continuous phase (PVA solution) [21]. The microdroplets traveled along the curved microchannels, and the PCL and CAMP solutions in the microdroplets began to mix with each other by diffusion and the swirling of the inner flow in the microdroplets [16]. During the solidification of fully mixed microdroplets by solvent evaporation, the solidified CAMPs occupy a volume in the microsphere owing to their dendritic growth. They can be removed by sublimation under vacuum freezing (−80 °C, 0.4 mTorr) or dissolution in an alcohol solution.

The In situ microfluidic device for the preparation of highly porous microspheres with controllable pore size was designed by the computer-aided design (CAD) program. The microfluidic flow-based device was adopted as a microfluidic tool to prepare highly porous PCL microspheres because it is relatively easy to control viscous solutions owing to their symmetry and narrow orifice. The designed microfluidic device had two inlets for the dispersed phase and one inlet for the continuous phase. The height of the microchannel was 150 μm, and the width of the microchannel at inlets was 300 μm. The length and width of the orifice of the designed microfluidic device were 300 and 100 μm. The microfluidic device had 20 curves to extend the movement of microdroplets in the microchannel to increase the mixing efficiency.

### 2.2. Microfluidic Device Fabrication

The master mold of the microfluidic device was fabricated by conventional photolithography. The negative photoresist (SU-8 2100, MICROCHEM, Newton, MA, USA) was dispensed on a 4-inch silicon wafer. The dispensed solution was homogenously spread using a spin coater with 3000 rpm for 30 s. Soft baking was conducted on a hot plate at 65 °C for 5 min and 95 °C for 30 min. The photoresist-coated wafer was selectively exposed through a photomask by a UV aligner. Consequently, post-exposure baking was performed to evaporate residual solvent (65 °C for 5 min and 95 °C for 12 min). The patterned wafer was dipped in a development solution (SU-8 developer, MICROCHEM, USA) for 15 min to remove the uncured polymer. Finally, the developed master mold was rinsed with isopropyl alcohol (IPA, DUCKSAN PURE CHEMICALS, Ansan, Gyeonggi-do, South Korea). The hard baking process (150 °C for 15 min) was conducted to enhance the attachment of the micropattern to the master mold. Additionally, the silanization (trichloro (1 h, 1 h, 2 h, 2 h-perfluorooctyl) silane, Sigma Aldrich, Seoul, South Korea) process was conducted on the fabricated master mold to prevent the attachment of the PDMS mold to the fabricated master mold. For the PDMS (Silgard 184, Dow Corning, Texas City, TX, USA) casting process, the PDMS prepolymer and the curing agent were mixed at a ratio of 10:1. The mixed solution was poured on the master mold and placed in a vacuum desiccator for 30 min to remove microbubbles from the PDMS solution. After removing microbubbles, the poured PDMS on the master mold was cured in the oven at 90 °C for 1 h. Finally, the cured PDMS block was detached from the master mold and permanently bonded with a glass slide using plasma treatment (PDC-32G, HARRICK PLASMA, Ithaca, NY, USA). To conserve the hydrophilicity of the microchannel after plasma treatment, PVA was coated on the surface of the microchannel. The PVA solution was injected into the fabricated microfluidic device. The microfluidic device filled with the PVA solution was placed in an oven at 90 °C for 5 min and at room temperature for 5 min. This same process was repeated 3 times.

### 2.3. Experimental Setup

All solutions were controlled independently by a syringe pump. The formation and behaviors of microdroplets in the microchannel were inspected by an optical inverted microscope (CSB-IH5, SAMWON Scientific Ind. Co., Ltd., Goyang, Gyeonggi-do, South Korea). The morphology of the prepared porous microspheres was observed by field-emission scanning electron microscopy (FE-SEM, S-4800, Hitachi High-Technology, Tokyo, Japan). Their diameter and pore size were analyzed using the image J program with FE-SEM images [13,22,23]. To observe the diffusion of PCL- and CAMP-involving microdroplets, a fluorescent dye (rhodamine dye) was used.

### 2.4. Preparation of Highly Porous Microspheres Using the In Situ Microfluidic System

To prepare dispersed phase solutions, PCL and CAMP solutions were prepared in DCM (dichloromethane, anhydrous ≥99.8%, Sigma Aldrich, Seoul, South Korea). The PVA solution was prepared by solubilizing PVA powder in deionized water at 90 °C. Briefly, 3 wt.% PCL and 20, 40, and 80 wt.% CAMP solutions as the dispersed phases were injected into the microfluidic device using syringe pumps, independently. In addition, 2 wt.% PVA solution was injected into the microfluidic device as the continuous phase solution [15]. As the dispersed phases, 0.5 and 0.5 mL/h for 1 (CAMP): 1 (3 wt.% PCL), 0.33 and 0.67 mL/h for 1:2, 0.25, and 0.75 mL/h for 1:3. The flow rates of the continuous phase flow (2 wt.% PVA) were 5, 10, and 20 mL/h for 20, 40, and 80 wt.% CAMP. PVA molecules of continuous phase were used as surfactants to prevent the coalescence of each microdroplet during the preparation process. The prepared porous microspheres were collected in 2 wt.% PVA solution under stirring at 300 rpm and placed at room temperature for 5 h to evaporate the residual solvent from the microspheres. The porous microspheres were washed with deionized water 3 times to remove the PVA solution on the surface of the porous PCL microspheres. Finally, CAMP in the microspheres was removed by sublimation under vacuum or dissolution in ethanol.

### 2.5. Cell Culture and Viability Assay

Fetal human dermal fibroblasts (HDFs) were purchased from Genlantis (USA). The cells were maintained at 37 °C in a Dulbecco’s modified Eagle medium (DMEM; Welgene, Gyeongsan-si, Gyeongsangbuk-do, South Korea) containing 10% fetal bovine serum (FBS; Biological Industries, Beit Haemek, Northern districts, Israel) and 1% penicillin-streptomycin (Gibco, Grand Island, NY, USA). The culture medium was changed every two days, and cells were passaged at subconfluency. Briefly, 10^4^ HDF cells were plated in each well of 96-well culture plates and stabilized for 24 h [24]. Consequently, the culture medium was replaced with 1 mL of DMEM with various concentrations of three different types of microspheres (1:1 (L), 1:2 (M), 1:3 (S)). After incubation for 24 h, cell viability was measured by an EZ-Cytox cell viability assay kit (DoGenBio, Seoul, South Korea) [25]. The cells were washed with phosphate-buffered saline (PBS) and then treated with 10 μL of the EX-Cytox solution and 100 μL of DMEM at 37 °C for 3 h according to the manufacturer’s instructions. After incubation, the absorbance was measured at 450 nm using a microplate spectrophotometer (SpectraMax Plus, Molecular Devices, San Jose, CA, USA). The absorbance values were normalized based on that of the negative control cell group (without microspheres) (*n* = 3).

### 2.6. Flow Cytometry Analysis of Inflammatory Protein Expression

Mouse leukemic monocyte macrophage RAW 264.7 cells (American Type Culture Collection, Manassas, VA, USA) were seeded (10^5^ per well) in each well of 6-well culture plates and sterilized for 24 h. The cells were treated with 3 different types of microspheres at 80 μg for 24 h and then harvested. The collected cells were fixed in 4% paraformaldehyde (PFA solution) for 4 h and washed with cold PBS. The washed cells were then treated with IL-6, TNF-α, and iNOS antibodies (Cell Signaling Technology, Denvers, MA, USA) for 12 h at 4 °C, washed with cold PBS and then treated with a FITC-conjugated secondary antibody (Santa Cruz, Dallas, TX, USA) for 1 h at room temperature. The inflammatory protein expression in the cells was quantified by flow cytometry (FACS). The cells without the microsphere treatment were used as a negative control, and the cells treated with LPS (1 μg/mL, Sigma Aldrich, Seoul, South Korea) were used as a positive control for inflammation.

### 2.7. In Vivo Biocompatibility: Animals and Implantation Procedures

Microspheres were injected subcutaneously in 11-week-old male Sprague Dawley rats (Koatech, Pyeongteak, Kyunggi-do, South Korea) weighing about 250 ± 50 g. Rats were fed with a laboratory diet, RO (Reverse Osmosis) water ad libitum. The animals were housed with 12 h dark and 12 h light cycles. The Animal Ethical Committee of the OSONG Medical Innovation Foundation approved the experimental protocol. All animals acclimated for 1 week prior to studies. The surgical procedure was performed under sterile conditions. The three types of microspheres were suspended in normal saline and implanted subcutaneously on the back of rats (3 types of polymer formulation; *n* = 4 implants/rat) under general anesthesia. Rats were sacrificed, and microsphere implants and surrounding tissues were explanted after 4 weeks.

### 2.8. Histological and Immunohistochemical Assay

The harvested animal tissues containing the microspheres were fixed with 10% buffered formalin fixative overnight prior to dehydration and paraffin embedding. The implants were cut into 5-μm-thick sections by microtome. General histologic assessment for the microspheres was based on hematoxylin and eosin (HE) and Masson’s trichrome (MT) staining according to the standard staining protocol. The local tissue effects and the inflammatory response at the implantation sites were evaluated using a slide scanner (Pannoramic SCAN II, 3D HISTECH, Budapest, Hungary) and observed and analyzed using an image analysis program, Case viewer (3D HISTECH, Budapest, Hungary). For the immunohistochemical staining of the in vivo samples, the deparaffinized sections were washed three times in cold PBS and incubated overnight at 4 °C with antibodies for iNOS (1:100, Cell Signaling Technology, Denvers, MA, USA) and TNF-α (1:100, Cell Signaling Technology, Denvers, MA, USA). After rinsing with cold PBS, the sections were incubated with the Alexa Fluor488-conjugated secondary antibody (Thermo Fischer, Waltham, MA, USA) in a humidified staining chamber for 1 h at room temperature. The nucleus of the cells was stained with DAPI (Thermo Fischer, Waltham, MA, USA). The observation and analysis of fluorescence tissues were performed using a confocal laser scanning microscope (Zeiss LSM 510, Carl Zeiss, Oberkochen, Germany).

## 3. Results and Discussion

The mixing of PCL and CAMP solutions was based on the diffusion and swirling of microdroplet flow along the microchannel. The boundaries of PCL and CAMP were observed immediately after microdroplet formation because the microdroplets have not yet been sufficiently mixed. However, the microdroplets were gradually mixed as they moved along the microchannel, and they were sufficiently mixed at the last section of the curved microchannel. The dispersion stability of CAMP in PCL microdroplets was tested using various solvents to investigate the best solvent for the porous microspheres. Chloroform, dichloromethane, ethyl acetate, dimethyl carbonate, and acetone were tested as solvents for PCL and CAMP. Porous microspheres were produced with these solvents by the conventional emulsification method. As a result, dichloromethane was selected as a solvent for our system (Figures S1 and S2).

**Figure 2:**
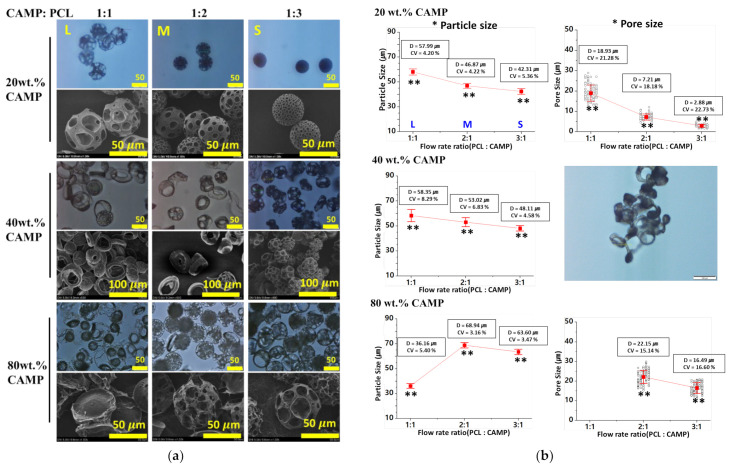
(**a**) Optical and SEM images of highly porous microspheres prepared by the in situ microfluidic system according to the flow rate ratio (CAMP: PCL) and the concentration of CAMP (20, 40, and 80 wt.%). As the dispersed phases, 0.5 and 0.5 mL/h for 1 (CAMP): 1 (3 wt.% PCL), 0.33 and 0.67 mL/h for 1:2, 0.25, and 0.75 mL/h for 1:3. The flow rates of the continuous phase flow (2 wt.% PVA) were 5, 10, and 20 mL/h for 20, 40, and 80 wt.% CAMP. (**b**) Porous microsphere size prepared under various conditions, such as 20, 40, and 80 wt.% CAMP concentrations in DCM and flow rate ratios using 3 wt.% PCL solution and various CAMP solutions (1:1, 1:2 and 1:3), (** *p* < 0.0001).

Figure 2a shows the optical and SEM images of highly porous microspheres under various conditions (PCL-to-CAMP flow rate ratio and concentration of CAMP). As the dispersed phase, 3 wt.% PCL solution and 20, 40, and 80 wt.% CAMP solutions were used, and the PCL-to-CAMP flow rate ratios of 1:1, 2:1, and 3:1 were investigated. Overall, the pore sizes of porous microspheres increased as their strength weakened with the increase in the flow rate of CAMP. Using 20 wt.% CAMP solution (L, M, and S), we observed that the pore sizes of porous microspheres can be controlled by the flow rate ratio of the PCL and CAMP solutions. With 80 wt.% CAMP solution, the microspheres became more porous and unstable than those obtained using 20 wt.% CAMP solution, and their structure collapsed with the increased flow rate of the CAMP solution (1:1 = PCL: CAMP). With 40 wt.% CAMP solution, donut-shaped microspheres were repeatedly formed at flow rate ratios of 1(PCL):1(CAMP) and 2:1. As the PCL flow rate increased, porous microspheres gradually tended to interconnect.

Figure 2b shows the diameters and pore sizes of the prepared microspheres with various concentrations (20, 40, and 80 wt.%) of CAMP and PCL-to-CAMP flow rate ratios (1:1, 2:1, and 1:1). The microfluidic device generated porous microspheres with narrow size distribution stably under all conditions. With 20 and 40 wt.% CAMP, the diameter of the porous microspheres decreased as the PCL flow rate increased because the solidified PCL volume was smaller than that of CAMP. Only with 80 wt.% CAMP (flow rate ratio = 1:1), the diameter of the porous microspheres decreased drastically because they were disrupted due to the weakened strength of their structure. The formed pore sizes of the porous microspheres were also measured and analyzed. With 20 and 80 wt.% CAMP, the pore sizes of the porous microspheres decreased as the ratio of the PCL flow rate increased. However, we excluded the microspheres prepared using 40 wt.% CAMP since they were non spherical. Additionally, in the case of 80 wt.% CAMP, the pore sizes of the porous microspheres were analyzed except at 1 (PCL): 1 (CAMP) because their structures were mostly disrupted at this condition. To improve the stability of their structures, the concentration of PCL must be increased (Figure S3).

**Figure 3:**
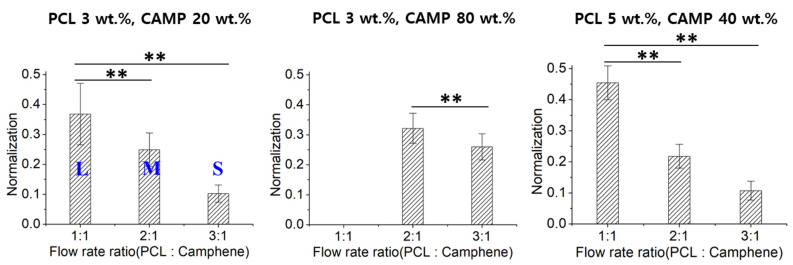
Normalized pore sizes of highly porous microspheres with respect to the flow rate ratios of PCL to CAMP (** *p* < 0.0001).

Since the diameters of the prepared porous microspheres were different according to the various conditions used, such as the ratio of flow rates and CAMP concentration, it is difficult to compare the pore sizes of porous microspheres. Therefore, the pore sizes of the microspheres were normalized with respect to the microsphere diameter (Figure 3). As a result, the pore sizes of porous microspheres significantly changed according to the ratio of flow rates after normalization.

**Figure 4:**
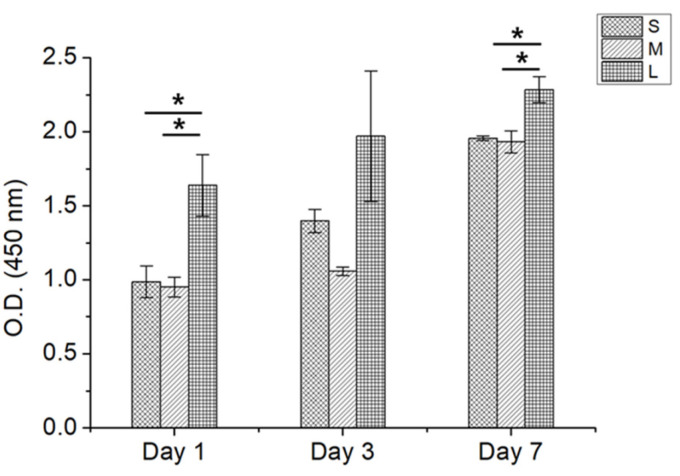
HDF cell proliferation results collected on highly porous PCL microspheres with various pore sizes after cell attachment for 1 week (* *p* < 0.05).

These results showed that the presented microfluidic system can control the pore size of highly porous microspheres in real time. We studied the effects of the controlled pore size of the highly porous microspheres when they were used for cell culture. To confirm the cell compatibility of the prepared PCL microspheres, cell viability tests were performed using HDF cells. The effects of microsphere treatment on the viability of cells were evaluated. The HDFs were cultured with highly porous microspheres of three different types (S: 3 wt.% PCL, 20 wt.% CAMP, a flow rate of 3 (PCL): 1 (CAMP), M: 3 wt.% PCL, 20 wt.% CAMP, a flow rate of 2 (PCL): 1 (CAMP) and L: 3 wt.% PCL, 20 wt.% CAMP, a flow rate of 1 (PCL): 1 (CAMP)) at various concentrations (0, 10, 20, 40, 80, 160, and 320 μg/mL) for 24 h, and the cell viability was measured by an EZ-Cytox cell viability assay kit. The microsphere-treated cells were highly viable for all highly porous microsphere types up to 320 μg/mL concentration and did not show any significant cell toxicity on HDF cells (Figure S4). To quantitatively analyze cell proliferation according to their pore sizes, CCK analysis was performed with HDF cells attached to the 3 different types of highly porous microspheres for 1 week (Figure 4).

From day 1 to 3, there was no significant difference between S and M microspheres for cell proliferation, but more cells were attached to the L microspheres. This result implies that the HDF cell could not penetrate into the pores of S and M microspheres only on the surface of the microsphere due to their seed size. After 7 days, the space inside the porous microspheres was saturated, and no significant cell proliferation difference was observed for S, M, and L microspheres. Furthermore, the number of L microspheres was larger than those of M and L microspheres at the same mass. Therefore, the cells gained not only an increased surface area but also additional space owing to the increased number of L microspheres.

**Figure 5:**
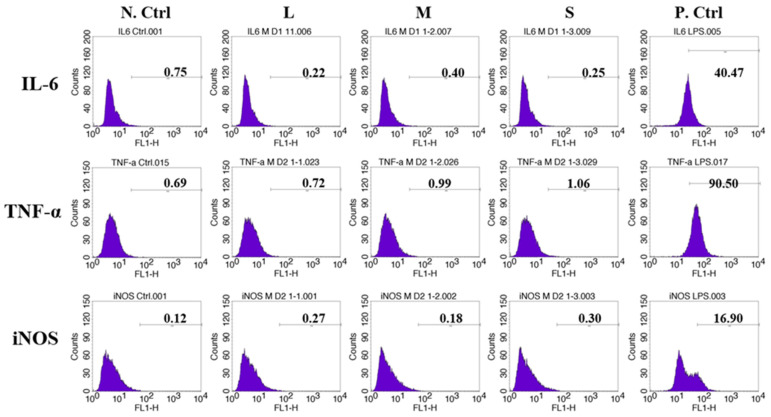
FACS histograms for the expression of inflammatory cytokines, IL-6, TNF-α, and iNOS, in RAW 264.7 cells treated with 3 different types of highly porous microspheres (L, M, and S) for 24 h. The cells without microspheres and LPS treatment were used as a negative control (N. Ctrl), and those treated with LPS (1 μg/mL) were used as a positive control (P. Ctrl).

The cellular expression of typical inflammation-related markers (IL-6, TNF-α, and iNOS) was analyzed by FACS. The populations of cells expressing IL-6, TNF-α, or iNOS proteins showed no inflammatory response for the three types of porous microspheres, as shown in the FACS histogram, compared with the positive control group (Figure 5).

**Figure 6:**
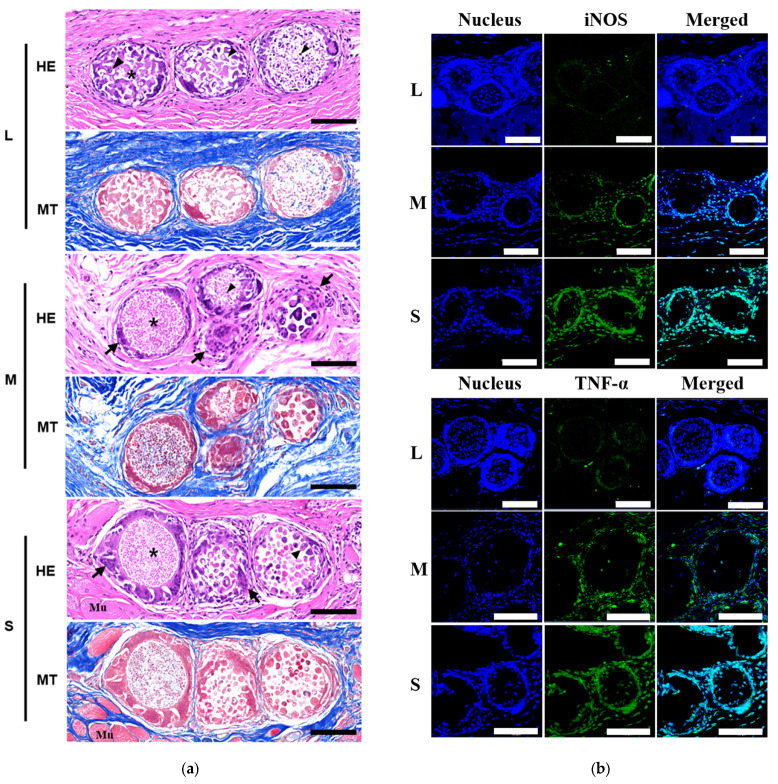
(**a**) Histological images of in vivo biocompatibility analysis for highly porous microspheres. The 3 different types of porous PCL microspheres (L, M, and S) were injected into the subcutaneous tissue of rats for 4 weeks and harvested after 4 weeks. *: PCL porous microspheres. Arrows: Inflammatory cells. Arrowheads: Cells infiltrated in PCL porous microspheres. Mu: The muscle tissue. HE: Hematoxylin–eosin staining. MT: Masson trichrome staining (Scale bar: 50 μm). (**b**) Immunofluorescence microscopic images of inflammation-related protein (iNOS and TNF-α) expression in the subcutaneous tissue of rats injected with highly porous PCL microspheres (scale bar: 100 μm).

To investigate the microsphere pore size effect on tissue regeneration, the three types of microspheres were subcutaneously injected into rats. Four weeks following implantation, tissue samples including the injected porous microspheres were harvested for histological and immune histological analysis. The HE and MT stain images were visualized to examine the biocompatibility and inflammatory response of the L, M, and S microspheres histologically (Figure 6a). In the L microsphere group, the surrounding connective tissues were in their native form, and no inflammation was observed. Some fibroblasts successfully migrated from the surrounding connective tissues into the porous microspheres via interconnected pores and adhered to the porous microspheres. The MT image showed collagens produced by fibroblasts in the porous microsphere. The M and S microspheres were surrounded by fibrous capsules and inflammatory cells. Contrary to the L microspheres, the cell migration and proliferation of fibroblasts into the porous structure were not observed in the case of the M and S microspheres.

Further, we performed immunohistochemical staining for the expression of inflammation-related markers, iNOS, and TNF-α, in the microsphere-implanted rat tissue matrix to evaluate the effects of the inflammatory response of highly porous microspheres (Figure 6b). The iNOS and TNF-α protein signals were very weak in the L microspheres, whereas the expression of both molecules showed strong signals in the M and S microspheres. The signals of iNOS and TNF-α proteins were stronger in the S microspheres compared with those in the M microspheres. The results showed that inflammation can be controlled by the microsphere pore size during tissue regeneration and that the porous microspheres with controllable pore sizes prepared by the in situ microfluidic system offer great potential to be used in tissue engineering.

## 4. Conclusions

An in situ microfluidic system for the preparation of highly porous PCL microspheres with controllable pore size was demonstrated. The in situ microfluidic system for the preparation of highly porous microspheres was fabricated by conventional photolithography and PDMS casting. Our in situ microfluidic system generated highly porous monodispersed PCL microspheres with precisely controlled pore size. The pore sizes of the microspheres can be controlled by adjusting the ratio of the flow rate of PCL (as the polymer phase) to that of CAMP (as the pore-generating material phase). With the increase in the flow rate of the CAMP solution, the porous microspheres had an increased porosity and decreased strength, disrupting their structures. Since the relationship between the stability and porosity of the microsphere are opposite, they should be balanced. In the case of 20 wt.% CAMP, stable porous microspheres with narrow size distribution were produced.

The porous microspheres of three pore sizes (S, M, and L) were evaluated using HDF cell viability (EX-Cytox cell viability assay), proliferation (CCK analysis), expression of inflammatory cytokines, including IL-6, TNF-α, and iNOS (FACS histogram) in vitro. The L microspheres showed a significantly increased proliferation of HDF cells compared with the S and M microspheres, while no significant differences were observed in other analyses. This result implied that the highly porous microsphere supplies a more excellent place for the proliferation of attached cells. Additionally, the highly porous microspheres were administrated into the subcutaneous tissue of rats to analyze the effects of the microsphere pore size. As the pore size of the injected porous microspheres increased, the inflammations of the tissue were significantly suppressed. The immunofluorescent assays of inflammation-related proteins (iNOS, TNF-α) from the harvested tissues of the porous microsphere-injected rats also supported these results. This means that a controlled porous microsphere provides an artificial implant, which relieved the immune response of injected tissue.

We envision that the pore size effect of implanted substrates is the key to controlling the inflammation of the tissue for tissue regeneration. This section may be divided into subheadings. It should provide a concise and precise description of the experimental results, their interpretation, as well as the experimental conclusions that can be drawn.

## Data Availability

The data used and/or analyzed during the current study are available from the corresponding author upon reasonable request.

## Supplementary Materials

The following supporting information can be downloaded at: https://www.mdpi.com/article/10.3390/pharmaceutics14112345/s1, Figure S1: Schematic of the solvent evaporation method for the preparation of porous PCL microsphere; Figure S2: (a) Optical and SEM images of porous microspheres by the emulsion method prepared under various solvent effects. Here, 3 wt.% PCL: 20 wt.% CAMP = 6:4 and 2 wt.% PVA in DI water were used; Figure S3: (a) Optical and SEM images of highly porous microspheres prepared by the microfluidic system according to the flow rate ratios using 5 wt.% PCL solution and 40 wt.% CAMP solution. (b) Diameter and pore size of porous microspheres with various flow ratios using 5 wt.% PCL solution and 40 wt.% CAMP solution as the dispersed phases (** *p* < 0.0001); Figure S4: HDF cell viability after highly porous microsphere treatment. HFD cells treated with 3 different types of microspheres (S, M, and L) at various concentrations for 24 h, and the viability analyzed using the EX-Cytox cell viability assay (*n* = 3).
