# Supplementary material for: Development of In Situ Microfluidic System for Preparation of Controlled Porous Microsphere for Tissue Engineering
Source: Pharmaceutics. 2022 Oct 30;14(11):2345. doi: 10.3390/pharmaceutics14112345 (PMC9693177; doi:10.3390/pharmaceutics14112345)
Supplement: Supplementary file 1 [file pharmaceutics-14-02345-s001.zip › pharmaceutics-1977265-supplementary.pdf]

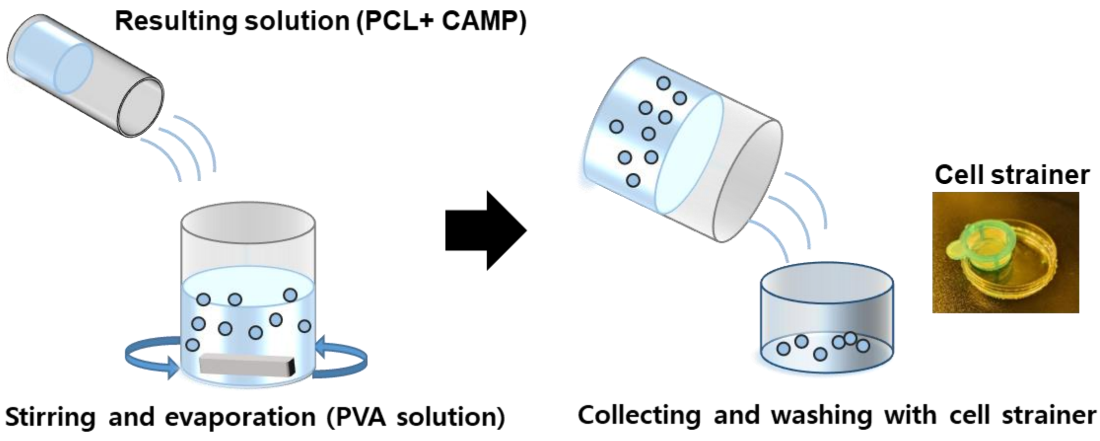

Figure S1. Schematic of the solvent evaporation method for the preparation of porous PCL microspheres.

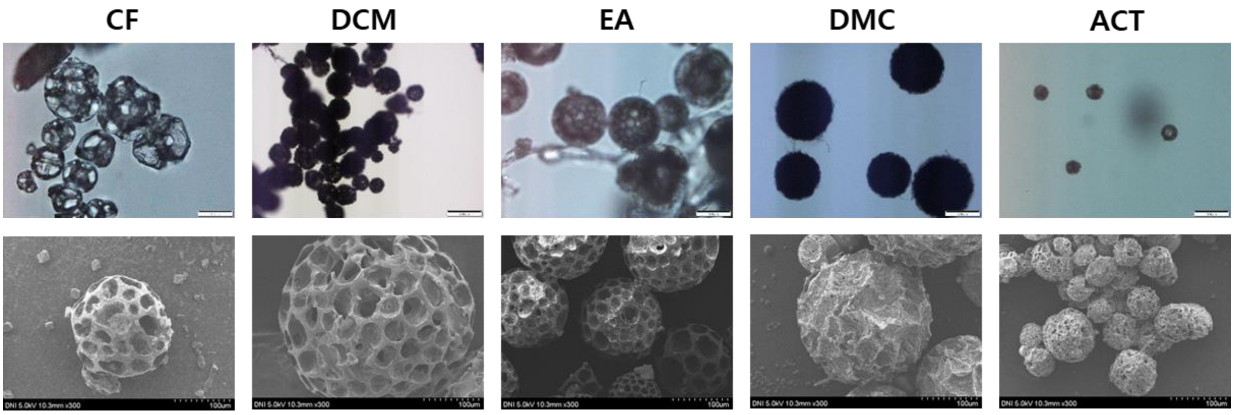

Figure S2. (a) Optical and SEM images of porous microspheres by the emulsion method prepared under various solvent effects. 3 wt.% PCL: 20 wt.% CAMP = 6:4 and 2 wt.% PVA in DI water were used.

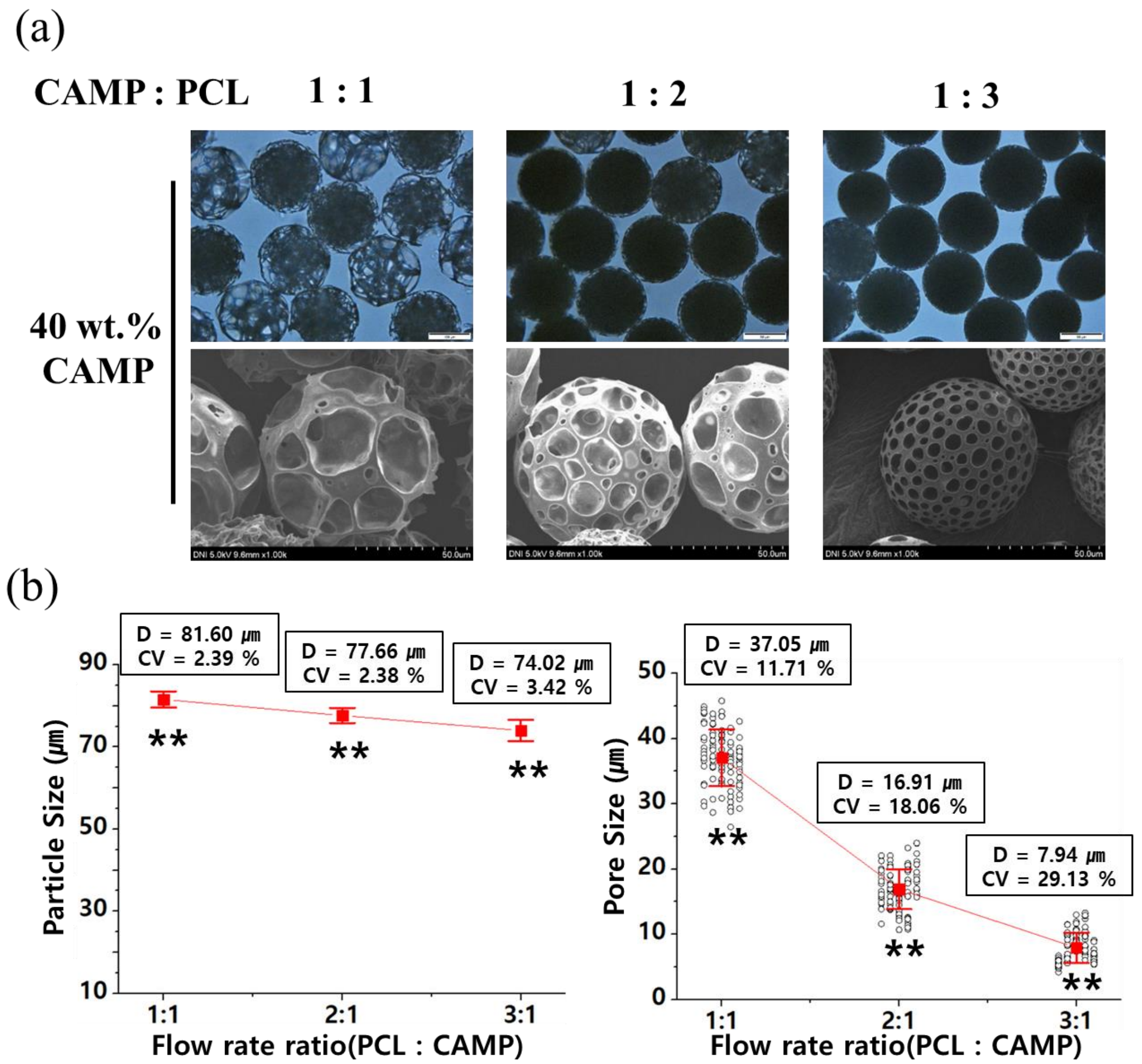

Figure S3. (a) Optical and SEM images of highly porous microspheres prepared by the microfluidic system according to the flow rate ratios using 5 wt.% PCL solution and 40 wt.% CAMP solution. (b) Diameter and pore size of porous microspheres with various flow ratios using 5 wt.% PCL solution and 40 wt.% CAMP solution as the dispersed phases (**$p < 0.0001$).

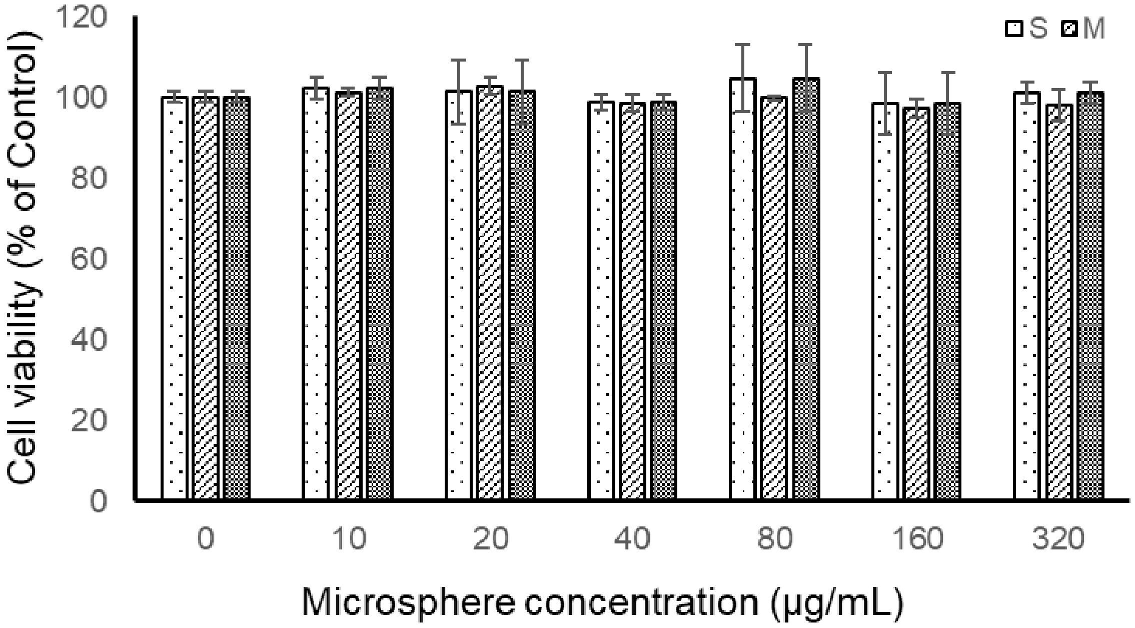

Figure S4. HDF cell viability after highly porous microsphere treatment. HFD cells treated with 3 different types of microspheres (S, M, and L) at various concentrations for 24 h, and the viability analyzed by the EX-Cytox cell viability assay (n = 3).
